# A Combination of Polybacterial MV140 and *Candida albicans* V132 as a Potential Novel Trained Immunity-Based Vaccine for Genitourinary Tract Infections

**DOI:** 10.3389/fimmu.2020.612269

**Published:** 2021-01-21

**Authors:** Leticia Martin-Cruz, Carmen Sevilla-Ortega, Cristina Benito-Villalvilla, Carmen M. Diez‐Rivero, Silvia Sanchez-Ramón, José Luis Subiza, Oscar Palomares

**Affiliations:** ^1^Department of Biochemistry and Molecular Biology, School of Chemistry, Complutense University, Madrid, Spain; ^2^Inmunotek, Alcalá de Henares, Madrid, Spain; ^3^Department of Clinical Immunology and IdISSC, Hospital Clínico San Carlos, Madrid, Spain; ^4^Department of Immunology, ENT and Ophthalmology, School of Medicine, Complutense University, Madrid, Spain

**Keywords:** dendritic cells, recurrent urinary tract infections (RUTIs), recurrent vulvovaginal candidiasis (RVVCs), trained immunity-based vaccines (TIbVs), *candida albicans* V132, polybacterial preparation MV140

## Abstract

Recurrent urinary tract infections (RUTIs) and recurrent vulvovaginal candidiasis (RVVCs) represent major healthcare problems with high socio-economic impact worldwide. Antibiotic and antifungal prophylaxis remain the gold standard treatments for RUTIs and RVVCs, contributing to the massive rise of antimicrobial resistance, microbiota alterations and co-infections. Therefore, the development of novel vaccine strategies for these infections are sorely needed. The sublingual heat-inactivated polyvalent bacterial vaccine MV140 shows clinical efficacy for the prevention of RUTIs and promotes Th1/Th17 and IL-10 immune responses. V132 is a sublingual preparation of heat-inactivated *Candida albicans* developed against RVVCs. A vaccine formulation combining both MV140 and V132 might well represent a suitable approach for concomitant genitourinary tract infections (GUTIs), but detailed mechanistic preclinical studies are still needed. Herein, we showed that the combination of MV140 and V132 imprints human dendritic cells (DCs) with the capacity to polarize potent IFN-γ– and IL-17A–producing T cells and FOXP3^+^ regulatory T (Treg) cells. MV140/V132 activates mitogen-activated protein kinases (MAPK)-, nuclear factor-κB (NF-κB)- and mammalian target of rapamycin (mTOR)-mediated signaling pathways in human DCs. MV140/V132 also promotes metabolic and epigenetic reprogramming in human DCs, which are key molecular mechanisms involved in the induction of innate trained immunity. Splenocytes from mice sublingually immunized with MV140/V132 display enhanced proliferative responses of CD4^+^ T cells not only upon *in vitro* stimulation with the related antigens contained in the vaccine formulation but also upon stimulation with phytohaemagglutinin. Additionally, *in vivo* sublingual immunization with MV140/V132 induces the generation of IgG and IgA antibodies against all the components contained in the vaccine formulation. We uncover immunological mechanisms underlying the potential mode of action of a combination of MV140 and V132 as a novel promising trained immunity-based vaccine (TIbV) for GUTIs.

## Introduction

Bacterial infections represent a major health-care problem and among them, urinary tract infections (UTIs) are one of the most common with a high incidence in women (1–4). UTIs are considered recurrent (RUTIs) with two or more symptomatic infections in less than six months or more than three per year (2, 5). Antibiotic treatment is the current therapy used for RUTIs, but its long-term use increases the risk to develop antibiotic resistance (3, 6). The overuse of antibiotics is also associated with dysregulation of normal vagina and gastrointestinal microbiota, which favor pathogen invasion and subsequent bacterial and fungal infection (1, 3, 7). Due to microbiota alterations, opportunistic pathogens such as *Candida albicans* colonize the vaginal tract generating vulvovaginal candidiasis (VVCs) (8–12). More than 70% of women worldwide suffer from VVCs (with maximum incidence between 20 and 40 years old) and around 5% experience recurrent infections (RVVCs), defined as four or more episodes per year (13–16). RVVCs require antifungal therapy to avoid recurrence and its overuse involves antimycotic resistances (17–19). Both RUTIs and RVVCs markedly diminish quality of life in women, with a negative impact at work and social life (20, 21). Therefore, there is an urgent need to develop novel strategies for concomitant and recurrent genitourinary tract infections (GUTIs), including RUTIs and RVVCs (1, 4, 16–18, 22).

Mucosal bacterial or fungal vaccines have been proposed as a new alternative approach to antibiotics and antifungals, respectively (23–25). These preparations containing soluble antigens or inactivated whole pathogens might well induce both specific immune responses and also non-specific immunomodulation. Sublingual administration of mucosal vaccines based on whole inactivated components has been recently reported as a suitable strategy to prevent and treat recurrent infections (24, 26–28). These preparations proposed as trained immunity-based vaccines (TIbVs) are able to induce long-lasting changes in innate immune cells, including dendritic cells (DCs), that enhance responses and protection against both the pathogens included in the vaccine formulation but also against unrelated pathogens by mechanisms involving immunological, metabolic and epigenetic reprogramming (23, 29–32). DCs survey the mucosal environment using innate pattern recognition receptors (PRRs), and are responsible for linking innate and adaptive immune responses (33, 34). Activation of DCs is linked to metabolic changes coupling the energetic needs with the functional activity (35, 36). Activation of DCs with microbial components promotes Warburg effect, a shift from oxidative phosphorylation to glycolysis and lactic fermentation under normal oxygen conditions (35, 37). This metabolic rewiring in DCs is driven at the molecular level by different mechanisms including activation of the axis protein kinase B (Akt)/mammalian target of rapamycin (mTOR)/hypoxia inducible factor-1α (HIF-1α) (35, 36).

MV140 (Uromune) is a polyvalent bacterial preparation based on whole heat-inactivated components used to prevent RUTIs (38). This sublingual vaccine is composed of bacteria causing the majority of RUTIs in Europe: 75% Gram-negative bacteria (25% *Escherichia coli*, 25% *Proteus vulgaris* and 25% *Klebsiella pneumoniae*) and 25% Gram-positive bacteria (*Enterococcus faecalis*) (26). Clinical data showed that MV140 significantly reduced the infection rates in patients suffering from RUTIs (39–43). MV140 acts directly on human DCs promoting maturation and activation through mechanisms depending on Syk- and MyD88-mediated signaling pathways, supporting the involvement of C-type lectin receptors (CLRs) and toll-like receptors (TLRs). MV140-induced downstream activation of NF-κB and p38 in DCs contribute to Th1/Th17 and IL-10 polarization whereas JNK and ERK are involved in Th1 and IL-10 immune responses, respectively (26). V132 is a preparation of heat-inactivated *C. albicans* that has been developed as a possible treatment of RVVCs. Given the tight relationship between RUTIs and RVVCs, a polymicrobial formulation, *via* combination of MV140 and V132, might well represent a suitable vaccine for both diseases.

In this study, we demonstrated that the combination of MV140 and V132 imprints human DCs with the capacity to generate potent IFN-γ– and IL-17A–producing T cells and FOXP3^+^ regulatory T (Treg) cells. Mechanistically, we showed that MV140/V132 activates inflammatory and mTOR pathways as well as metabolic and epigenetic reprogramming in human DCs. *In vivo*, sublingual immunization of BALB/c mice with MV140/V132 enhances the proliferation of splenic CD4^+^ T cells upon *in vitro* stimulation with the antigens contained in the formulation and with phytohaemagglutinin (PHA). Sublingual immunization with MV140/V132 also induces the generation of specific IgG and IgA antibodies against all the components contained in the formulation. Overall, we show for the first time molecular mechanisms that might be involved in the mode of action of MV140/V132 as a novel promising trained immunity-based vaccine (TIbV) for GUTIs.

## Materials and Methods

### Media and Reagents

RPMI 1640 (Lonza) supplemented with 10% fetal bovine serum, 100 μg/ml normocin, 50 μg/ml penicillin-streptomycin, 1% non-essential amino acids, 1% MEM-vitamins and 1 mM sodium pyruvate. MV140 (Uromune) composed of whole heat-inactivated bacteria (25% *E. coli*, 25% *P. vulgaris*, 25% *K. pneumoniae* and 25% *E. faecalis*), V132 composed of whole heat-inactivated *C. albicans*, MV140/V132 (combination of MV140 and V132) and control excipients (negative control, containing all excipients without bacteria and yeast) were provided by Inmunotek S.L. Inhibitors for histone methyltransferase 5′-deoxy-5′-(methylthio) adenosine (MTA) and histone demethylase (Pargyline) (Sigma-Aldrich) were used for the inhibition experiments.

### Generation of Human Monocyte-Derived Dendritic Cells and Purification of Naïve CD4^+^ T Cells

Peripheral blood mononuclear cells (PBMC) from buffy coats of healthy donors were obtained by using Ficoll density gradient centrifugation (800*g*, 20 min). Monocytes were isolated from total PBMC using anti-CD14 microbeads and cultured for 6 days with RPMI medium containing 100 ng/ml of IL-4 and GM-CSF (PeproTeck) to generate immature human monocyte-derived DCs (hmoDCs). The purity and phenotype of monocytes and generated immature hmoDCs were analyzed by flow cytometry with lineage-specific markers. Peripheral blood naive CD4^+^ T cells were isolated using the “Naive CD4^+^ T Cell Isolation Kit” (Miltenyi Biotec), according to manufacturer’s protocol.

### Cell Cultures

Immature hmoDCs (10^6^ cells per ml) were stimulated with control excipients (containing all excipients except the microbial components), V132 (3 FTU, Formazan Turbidity Units, per ml), MV140 (10^7^ bact. per ml) or MV140/V132 (MV140, 10^7^ bact. per ml, and V132, 3 FTU per ml) for 18 h. Subsequently, cells were collected and centrifuged. Cell pellets were used to analyze their phenotype by flow cytometry and cell-free supernatants were used to quantify IL-6, TNF-α, IL-23, IL-1β, IL-10, and TGF-β1 by ELISA. For inhibition experiments, hmoDCs were preincubated for 1 h with MTA (0.5 mM) or Pargyline (3 μM) (or their vehicle control, DMSO) prior to activation. Then, the cells were stimulated with stimuli for 18 h in the presence of the corresponding inhibitors to quantify TNF-α, IL-23, and IL-10 by ELISA. Cell viability was analyzed in all the cases by trypan blue exclusion with a light microscope.

### Flow Cytometry

The following anti-human monoclonal antibodies (mAbs) were used for flow cytometry: fluorescein isothiocyanate (FITC)-conjugated anti-HLA-DR; phycoerythrin (PE)-conjugated anti-CD86; and allophycocyanin (APC)-conjugated anti-CD83 (Miltenyi Biotec). APC-conjugated anti-CD3; and Alexa Fluor 488-conjugated anti-IFN-γ (BD Pharmigen). PE-conjugated anti-IL-10; Alexa Fluor 488-conjugated anti-IL-17A; peridinin-chlorophyll-protein (PerCP)-conjugated anti-CD4; Alexa Fluor 488-conjugated anti-FOXP3; PE-conjugated anti-CD127; and APC-conjugated anti-CD25 (Biolegend). Cells were washed with PBS/EDTA 2 mM/0.5% BSA and stained for 15 min at room temperature in the darkness. For analysis of FOXP3 expression in human T cells primed with hmoDCs, cells were first subjected to surface staining with anti-human CD127-PE, CD4-PerCP, and CD25-APC antibodies. After fixation and permeabilization, cells were stained with anti-human FOXP3-Alexa Fluor 488, according to manufacturer’s recommendations. For each staining, the corresponding isotype controls (IgG2A-FITC, IgG1-Alexa Fluor 488, IgG1-PE, IgG2A-PerCP, or IgG1-APC) were also assayed. Flow cytometry analysis was performed in a FACSCalibur in the Cytometry and Fluorescence Microscopy Unit at Complutense University of Madrid.

### Cytokine Quantification

The cytokine production of IL-6, TNF-α, IL-1β, IL-10, IFN-γ, IL-5 (BD Biosciences), IL-23 (Invitrogen), IL-17A (Mabtech), and TGF-β1 (R&D System) was quantified by sandwich ELISA in cell-free supernatants following the manufacturer’s instructions.

### Co-Culture Experiments

HmoDCs were co-cultured with purified allogeneic naïve CD4^+^ T cells (1:5 DC/T cell) for 3 days in the presence of control excipients, V132 (3 FTU per ml), MV140 (10^7^ bact. per ml) or MV140/V132 (MV140, 10^7^ bact. per ml, and V132, 3 FTU per ml). IFN-γ, IL-5, IL-17A, and IL-10 were quantified in cell-free supernatants by ELISA. For proliferation assays, purified allogeneic naive CD4^+^ T cells were labeled with CFSE (Molecular Probes) prior to co-culture with immature hmoDCs. Proliferation was assessed by measuring CFSE dilution on labeled CD4^+^ T cells by flow cytometry. For intracellular staining, the primed CD4^+^ T cells were washed and restimulated with 25 ng/ml of PMA plus 1 μg/ml of ionomycin for 6 h and 10 μg/ml of Brefeldin A for the last 4 h. Cells were fixed and permeabilized with Cytofix/Cytoperm (BD Biosciences) according to the manufacturer’s instructions. The cells were stained with the combination of fluorochrome-labeled mAbs to IFN-γ, IL-10, and IL-17A.

### Western Blot Analysis

HmoDCs (10^6^ cells per ml) were treated with control excipients, V132 (3 FTU per ml), MV140 (10^7^ bact. per ml) or MV140/V132 (MV140, 10^7^ bact. per ml, and V132, 3 FTU per ml). After 30 min at 37°C, cells were harvested and lysed with RIPA buffer (ThermoFisher scientific) in presence of Protease/Phosphatase Inhibitor Cocktail (Cell Signaling) for 30 min at 4°C vortexing every 10 min. Lysates were clarified by centrifugation at 10,000*g* for 15 min at 4°C. Protein quantification was performed with Micro BCA Protein Assay Kit (Pierce) and samples with equal amounts of total protein were resolved in 10% SDS-polyacrylamide gel electrophoresis (SDS–PAGE). Proteins were then transferred to nitrocellulose membrane (BioRad). The membrane was incubated with the primary antibodies: phospho-ERK1/2 (Thr202/Tyr204), phospho-SAPK/JNK (Thr183/Tyr185), phospho-p38 MAPK (Thr180/Tyr182), phospho-IKKα (Ser176)/IKKβ (Ser177), or phospho-Akt (Ser473) (1:1000, Cell Signaling), phospho-p70 S6 Kinase (Thr389) (1:750; Cell Signaling), and β-actin (1:15000, Sigma-Aldrich); and goat anti-rabbit (1:4000, BioRad) or goat anti-mouse (1:2500, Pierce) conjugate with horseradish peroxidase as a secondary antibody. The signal was developed with Clarity Western ECL Substrate (Bio-Rad) and detected in a Fujifilm LAS-3000 developer.

### Metabolic Studies

HmoDCs were stimulated with control excipients, V132 (3 FTU per ml), MV140 (10^7^ bact. per ml) or MV140/V132 (MV140, 10^7^ bact. per ml, and V132, 3 FTU per ml) for 18 h. Glucose concentration in cell-free supernatants was determined by using the Glucose (GO) Assay Kit (Sigma-Aldrich). The metabolic rate was derived mathematically in percentage of medium without hmoDCs (2 mg/ml). Lactate production in cell-free supernantants was determined by using the colorimetric L-Lactate Assay kit (Sigma-Aldrich). The mitochondrial membrane potential was measured using MitoTracker Red Mitochondrion-Selective Probe kit (ThermoFisher Scientific) following the manufacturer’s instructions with minor modifications. Briefly, mitochondrial activation was measured using MitoTracker Red CMXROS (250 nM) for 30 min at 37°C after 18 h of treatment with the corresponding stimulus. The fluorescence of the probe was analyzed using FLUOstar OPTIMA fluorescence reader (BMG LabTech).

### RNA Extraction, cDNA Synthesis, and Quantitative Real-time RT-PCR

RNA was isolated from harvested cells using an RNeasy mini kit (Qiagen) and cDNA generated using a PrimeScript RT reagent Kit (Takara) according to manufacturer’s instructions. Real-time quantitative PCR was performed on cDNA using FastStart Universal SYBR Green Master (Rox) (Roche). The sequences of the used pair primers were: cyclooxygenase II (COXII) (forward, CTATCCTGCCCGCCATCATC; reverse, GGGATCGTTGACCTCGTCTG), glyceraldehyde-3-phosphate dehydrogenase (GAPDH) (forward, CTGCACCACCAACTGCTTAGC; reverse, TCATGTTCTGGAGAGCCCCG), Hypoxia-inducible factor 1α (HIF-1α) (forward, TCGCATCTTGATAAGGCCTCT; reverse, ACAAAACCATCCAAGGCTTTCA), and Elongation Factor 1α (EF1α) (forward, CTGAACCATCCAGGCCAAAT; reverse, GCCGTGTGGCAATCCAAT). Samples were run on a real-time PCR system (ABI Prism 7900 HT; Applied Biosystems). Data were normalized to EF1α and displayed as arbitrary units calculated as 2^−ΔCT^ values multiplied by 10^4^. ΔCT was defined as the difference between the cycle threshold value for the gene of interest and EF1α. The mitochondrial/nuclear DNA content was calculated by using the formula: 2^-ΔCT^ mitochondrial COXII/2^−ΔCT^ nuclear GAPDH.

### Sublingual Immunization of Mice With MV140/V132 or Control Excipients: Quantification of CD4^+^ T Cell Proliferation of *In Vitro* Stimulated Splenocytes With V132, MV140, MV140/V132, PHA, or Control Excipients, and Induced Serum-Specific Antibody Levels

BALB/c mice (6-weeks-old) were immunized four times with 20 μl of MV140/V132, MV140 alone, V132 alone (300 FTU per ml) or control (all excipients without bacteria and yeast) by sublingual administration every 7 days and killed 7 days after the last immunization. Sublingual administration was performed under anaesthesia (ketamine, 100 mg/kg and xylacine, 5 mg/kg) to ensure proper delivery. Spleens were used to prepare single cell suspensions following conventional protocols. Cells were labeled with CFSE (5 mM, Life technologies) and stimulated *in vitro* with V132 (6 FTU per ml), MV140 (6 FTU per ml), MV140/V132 (MV140, 3 FTU per ml, and V132, 3 FTU per ml), phytohaemagglutinin (PHA, 5 µg/ml, Sigma-Aldrich) or control excipients for 5 days. Proliferation of CFSE-labeled CD4^+^ T cells was monitored by flow cytometry. Serum specific IgG, IgA, IgG1, and IgG2a for *C. albicans*, *K. pneumoniae*, *E. coli*, *E. faecalis*, and *P. vulgaris* induced after sublingually immunization were determined by ELISA. Briefly, 96-well non-tissue culture-treated plates were pretreated with poly-L-lysine (Sigma-Aldrich) for 1 h under UV light and coated with each of the heat-inactivated whole cell bacteria or yeast (300 FTU per ml) overnight at 4°C, and, subsequently, incubated with mouse serum dilutions for 2 h at room temperature. Specific immunoglobulins were detected with biotin rat anti-mouse IgA and IgG (both from Sigma-Aldrich). Signal was developed by incubation with streptavidin-horseradish peroxidase (HRP) (Sigma-Aldrich). Peroxidase activity was revealed by the addition of o-phenylenediamine dihydrochloride (Sigma-Aldrich) and the reaction was stopped with HCl 1 N. Plates were read on an ELISA reader at 490 nm (Triturus Elisa, Grifols). Animals were maintained in Biolab S.L. under the conditions stabilized by the R.D. 53/2013 and in accordance with the international GLP normative, RD 223/83 and ISO 10993-2:2007. The tests are performed in accordance with official methods, European Pharmacopoeia, USP Pharmacopoeia, OECD Guideline, UNE EN-ISO, and specific methodologies developed for R&D.

### Statistics

All the data are expressed as means ± s.e.m. of the indicated parameters. Paired or unpaired Student’s t-test used for statistical analysis were performed using GraphPad Prism software, version 6.0. Significance was defined as *P<0.05, **P<0.01 and ***P<0.001.

## Results

### MV140/V132-Activated Human Dendritic Cells Display Significantly Higher Levels of CD83, Pro-Inflammatory Cytokines, and IL-10 Than Dendritic Cells Stimulated With the Individual Components

**Figure 1:**
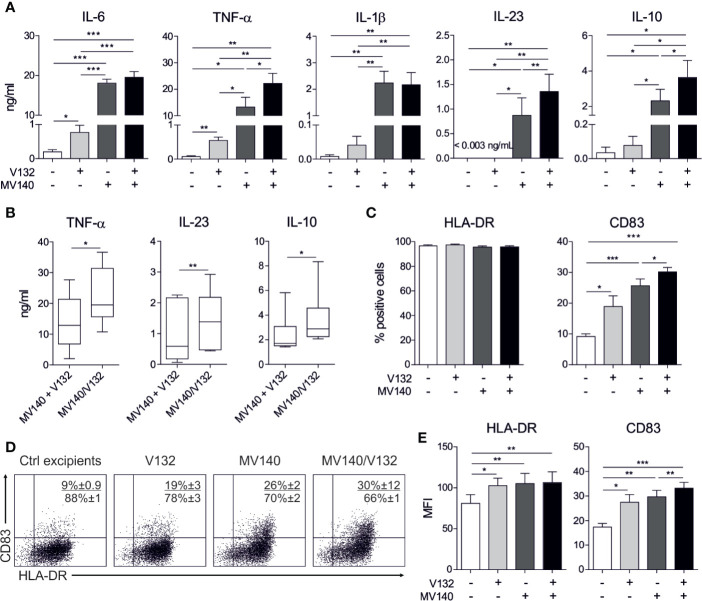
MV140/V132 induces hmoDCs maturation and production of pro-inflammatory cytokines and IL-10. **(A)** Cytokine production after stimulation of hmoDCs with control excipients (containing all excipients except the bacteria and yeast), V132, MV140, and MV140/V132 for 18 h quantified in cell-free supernatants by ELISA. **(B)** Cytokine levels of V132-treated hmoDCs plus cytokine levels of MV140-treated hmoDCs compared with cytokine levels of MV140/V132-stimulated hmoDCs. **(C)** Percentage of CD83- and HLA-DR-positive cells after stimulation with control excipients, V132, MV140 and MV140/V132 for 18 h. **(D)** Flow cytometry representative dot plots for the expression of surface markers CD83 and HLA-DR after stimulation of hmoDCs with the indicated stimulus. **(E)** Mean Fluorescence Intensity (MFI) values for CD83 and HLA-DR. Results are mean ± s.e.m. of 6-8 **(A)**, 6 **(B)**, and 7-8 **(C, E)** independent experiments. Paired Student *t* test, *P < 0.05, **P < 0.01, and ***P < 0.001.

To assess the capacity of the combination of the polyvalent bacteria preparation MV140 and *C. albicans* V132 to modulate the phenotype and function of human DCs, we treated human monocyte-derived DCs (hmoDCs) from healthy donors with V132, MV140, MV140/V132 or control excipients. HmoDCs treated with V132 produced significantly higher levels of IL-6 and TNF-α, and a slight increase in the production of IL-1β and IL-10 than control excipients. IL-23 levels were no detectable in cell-free supernatants from V132-treated hmoDCs (**Figure 1A**). MV140 induced significant production of IL-6, TNF-α, IL-1β, IL-23, and IL-10 compared to control treatment, as previously described (26), and also compared to V132 (**Figure 1A**). MV140/V132-activated hmoDCs induced significantly higher levels of pro-inflammatory cytokines and IL-10 than excipients- or V132-activated hmoDCs (**Figure 1A**). Interestingly, MV140/V132 stimulation induced significantly higher levels of TNF-α, IL-23, and IL-10 than MV140 in hmoDCs (**Figure 1A**). To test whether simultaneous stimulation of hmoDCs with MV140/V132 enhance the production of TNF-α, IL-23, and IL-10 in a synergistic manner, cytokine levels induced by MV140/V132 showed in **Figure 1A** were directly compared to those resulting from the addition of the cytokine levels produced by activation with MV140 and V132 separately (**Figure 1A**). MV140/V132-activated hmoDCs induced significantly higher levels of TNF-α, IL-23, and IL-10 than the corresponding cytokine levels produced by V132- plus MV140-treated hmoDCs, supporting synergistic cooperation in the simultaneous stimulation with the combination of MV140 and V132 (**Figure 1B**). We did not detect significant differences in the levels of TGF-β1 among any of the assayed conditions (**Supplementary Figure 1A**). Although there were no significant differences in the percentage of cells expressing HLA‐DR (**Figures 1C, D**), the expression levels (MFI values) were significantly increased after V132, MV140 and MV140/V132 stimulation compared to excipients-treated cells, without significant differences among them (**Figure 1E**). V132, MV140 and MV140/V132 significantly increased the expression of the co-stimulatory molecule CD83 in hmoDCs compared to control excipients (**Figures 1C, D**). MV140 and MV140/V132 induced higher expression of CD83 than V132 without significant differences (**Figures 1C, D**). Both the percentage of positive cells and the expression levels of CD83 were significantly higher in hmoDCs stimulated with MV140/V132 than MV140, indicating that the combination of these stimuli in a single formulation significantly enhance the maturation of hmoDCs (**Figures 1C, E**). The expression of CD86 was significantly higher in hmoDCs stimulated with V132 than the other stimuli, but no differences were observed after MV140 and MV140/V132 stimulation compared to control excipients (**Supplementary Figures 1B–D**).

### MV140/V132-Activated Human Dendritic Cells Generate Potent IFN-γ- and IL-17A–Producing T Cells and FOXP3^+^ Treg Cells

**Figure 2:**
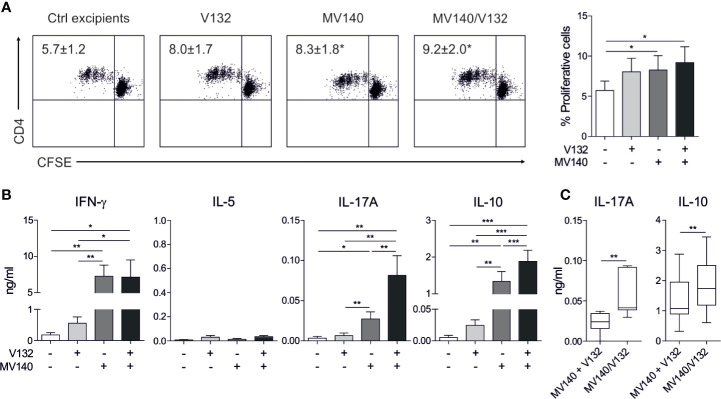
MV140/V132-activated hmoDCs induce T cell proliferation and Th1, Th17, and IL-10 producing T cells. **(A)** Representative dot plots of proliferating CFSE-labeled allogeneic naive CD4^+^ T cells after 3 days of co-culture with control excipients-, V132-, MV140-, and MV140/V132-stimulated hmoDCs and the graph with the frequency of proliferating cells (right side). **(B)** Cytokines produced by allogeneic naive CD4^+^ T cells primed by hmoDCs in the presence of the indicated stimulus after 3 days quantified in cell-free supernatants by ELISA. **(C)** Levels of IL-17A and IL-10 for V132- plus MV140-stimulated co-cultures compared to MV140/V132-stimulated co-cultures. Results are mean ± s.e.m. of 5 **(A)**, and 8 to 12 **(B, C)** independent experiments. Paired Student *t* test, *P < 0.05, **P < 0.01, and ***P < 0.001.

**Figure 3:**
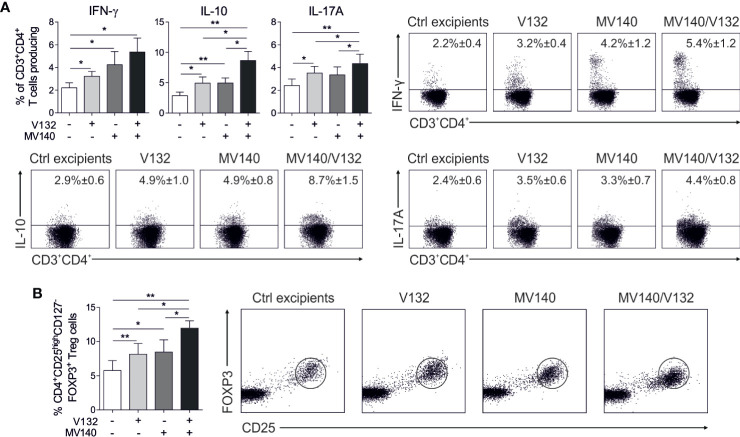
MV140/V132-activated hmoDCs generate IFN-γ-, IL-17A-, and IL-10-producing T cell production as well as FOXP3^+^ Treg cells. **(A)** Percentage of CD3^+^CD4^+^ T cells producing IFN-γ, IL-10 and IL-17A generated after 3 days of co-culture with control excipients-, V132-, MV140-, and MV140/V132-stimulated hmoDCs as determined by intracellular staining and flow cytometry analysis. Representative dot plots are shown for the intracellular staining after flow cytometry analysis. **(B)** Percentage of induced CD4^+^CD25^high^CD127^-^FOXP3^+^ Treg cells gated on CD4^+^ T cells after 3 days of co-culture with allogeneic control excipients-, V132-, MV140-, and MV140/V132-activated hmoDCs. Results are mean ± s.e.m. of 7-8 **(A)**, and 5 **(B)** independent experiments. Paired Student *t* test, *P < 0.05, and **P < 0.01.

To determine the capacity of human DCs treated with the combination of MV140 and V132 to polarize CD4^+^ T cell responses, we performed co-culture experiments. HmoDCs stimulated with MV140 and MV140/V132 induced a significantly higher percentage of proliferating allogeneic CD4^+^ T cells than excipients-treated hmoDCs (**Figure 2A**). V132-activated hmoDCs were also able to stimulate proliferation of CD4^+^ T cells but significant changes were not detected when comparing with excipients-treated hmoDCs (**Figure 2A**). HmoDCs activated with MV140 or MV140/V132 generated T cells producing significantly higher levels of IFN-γ, IL-17A, and IL-10 than excipients- or V132-stimulated hmoDCs (**Figure 2B**). We did not detect IL‐5 production in any of the assayed conditions (**Figure 2B**). Interestingly, MV140/V132-activated hmoDCs generated T cells producing significantly higher levels of IL-17A and IL-10 than MV140-activated hmoDCs (**Figure 2B**). To assess potential synergistic effects, the levels of IL-17A and IL-10 produced by T cells generated by MV140/V132-activated hmoDCs (**Figure 2B**) were compared to those resulting from the addition of these cytokine levels in MV140- and V132-stimulated conditions separately, shown in **Figure 2B**. IL-17A and IL-10 production was significantly higher when T cells were primed by hmoDCs activated with MV140/V132 than the sum of the cytokine levels produced by T cells generated by V132- plus MV140-activated hmoDCs, thus supporting synergistic effects of MV140/V132 at the T cell level (**Figure 2C**). To verify these data at the single cell level, we performed intracellular staining experiments. The percentages of IFN-γ–, IL-10–, and IL-17A–producing CD4^+^ T cells generated by hmoDCs treated with V132, MV140 or MV140/V132 were significantly higher than those produced by T cells generated by excipients-treated hmoDCs (**Figure 3A**). Supporting our results, MV140/V132-activated hmoDCs generated a significantly higher percentage of IL-10– and IL-17A–producing T cells than MV140-treated cells (**Figure 3A**). To investigate the capacity of activated hmoDCs to induce Treg cells, we also analyzed the generation of FOXP3^+^ Treg cells in allogeneic co-culture experiments. V132-, MV140-, and MV140/V132-treated hmoDCs induced significantly higher percentages of CD4^+^CD25^high^CD127^-^FOXP3^+^ Treg cells than excipients-treated hmoDCs (**Figure 3B**). Remarkably, the number of FOXP3^+^ Treg cells generated by MV140/V132-activated hmoDCs was significantly higher than those induced by hmoDCs stimulated with V132 or MV140 alone (**Figure 3B**).

### MV140/V132 Stimulation in Human Dendritic Cells Activates Mitogen-Activated Protein Kinases, Nuclear Factor-κB, and mTOR Signaling Pathways

**Figure 4:**
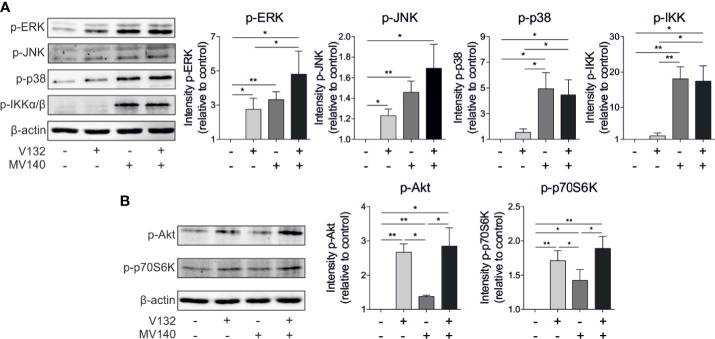
Activation of MAPKs, NF-κB, and mTOR signaling pathways in hmoDCs stimulated with control excipients, V132, MV140, or MV140/V132. **(A, B)** Western blot analysis of protein extracts from hmoDCs stimulated for 30 min in the indicated conditions. Quantification of the reactive phosphorylated bands by scanning densitometry. β-actin was used as a loading control. One representative example is shown. Results are mean ± s.e.m. of four to seven independent experiments. Paired Student *t* test, *P < 0.05, and **P < 0.01.

MV140 triggers CLRs and TLRs in human DCs, which culminate in the activation of mitogen-activated protein kinases (MAPKs)- and nuclear factor-κB (NF-κB)-mediated signaling pathways (26). *C. albicans* activates a complex network of PRRs that also lead to the subsequent activation of MAPKs and NF-κB (44). Our data revealed that V132 rapidly induced activation of the MAPK extracellular signal-regulated kinases 1/2 (ERK1/2) (phosphorylation of Thr202/Tyr204) and the MAPK c-Jun NH_2_-terminal kinase (JNK) (phosphorylation of Thr183/Tyr185) in hmoDCs without activation of MAPK p38 (phosphorylation of Thr108/Tyr182) (**Figure 4A**). MV140 alone induced a significantly higher phosphorylation of p38 and IKKα/β than V132 alone in hmoDCs without significant changes observed for ERK or JNK compared to V132 alone (**Figure 4A**). V132 alone induced significant phosphorylation of ERK and JNK compared to unstimulated cells without changes in p38 or IKKα/β. Interestingly, MV140/V132 induced a significantly higher phosphorylation of ERK, p38, and IKKα/β than V132 (**Figure 4A**). However, a slight but non-significant increment in the activation of ERK and JNK was observed after MV140/V132 stimulation compared with MV140 alone without differences in the activation of p38 or IKKα/β (**Figure 4A**). Sensing of *C. albicans* β-glucan by dectin-1 induces the activation of mTOR signaling pathway (31), however, the implication of mTOR pathway in the mechanism of action of MV140 remains unknown. V132, MV140 and MV140/V132 induced the activation of the mTOR pathway as determined by the phosphorylation of the upstream activator Akt (Ser473) and the downstream substrate p70S6 kinase (Thr389) (**Figure 4B**). V132 and MV140/V132 stimulation induces a similar and significantly higher phosphorylation of Akt and p70S6K than MV140 in hmoDCs (**Figure 4B**).

### MV140/V132 Induces Metabolic and Epigenetic Reprogramming in Human Dendritic Cells

**Figure 5:**
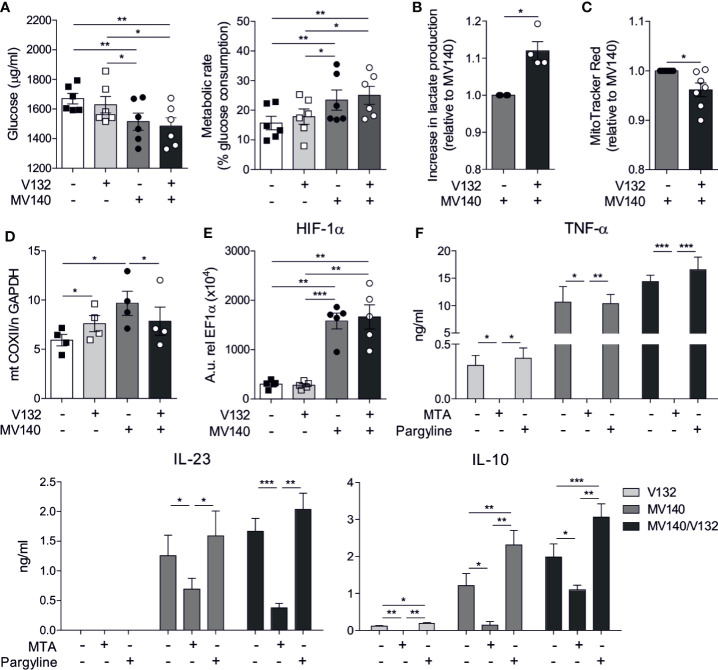
MV140/V132 induces metabolic and epigenetic reprogramming in hmoDCs. **(A)** Glucose consumption by hmoDCs treated with control excipients, V132, MV140 and MV140/V142 after 18 h and calculated metabolic rate. **(B)** Increase in lactate production in cell-free supernatants from MV140/V132-stimulated hmoDCs relative to MV140-activated hmoDCs. **(C)** Relative changes in fluorescence intensity of stimulated hmoDCs stained with MitoTracker Red. **(D)** Mitochondrial mass determination as the ratio of mitochondrial (mt) and nuclear (n) DNA as expressed by mt COXII/n GAPDH. **(E)** Messenger RNA expression levels of HIF-1α gene in hmoDCs stimulated for 18 h. Arbitrary units (A.u.) are 2^−ΔCT^ values multiplied by 10^4^, with ΔCT defined as the difference between the cycle threshold value for the gene of interest and EF1α. **(F)** Cytokine production by V132-, MV140-, and MV140/V132-activated hmoDCs in the presence of 5′-methylthioadenosine (MTA) or pargyline as inhibitors of histone methyltransferases and demethylases, respectively. Results are mean ± s.e.m. of 6 **(A)**, 4 **(B)**, 7 **(C)**, 4 **(D)**, 5 **(E)**, and 4-7 **(F)** independent experiments. Paired Student *t* test, *P < 0.05, **P < 0.01, and ***P < 0.01.

We studied metabolic changes in hmoDCs stimulated with V132, MV140 or the combination of MV140 and V132. A slight non-significant increase in the consumption of glucose and metabolic rate was observed in hmoDCs after V132 stimulation compared to excipients-treated hmoDCs (**Figure 5A**). In contrast, the stimulation of hmoDCs with MV140 or MV140/V132 significantly enhanced the consumption of glucose from the culture medium and, therefore, the metabolic rate compared to excipients- or V132-treated hmoDCs, without significant differences among them (**Figure 5A**). However, MV140/V132-activated hmoDCs significantly increased lactate production relative to MV140-stimulated hmoDCs, suggesting that the combination of MV140 and V132 is able to enhance Warburg effect in human DCs (**Figure 5B**). In the same line, hmoDCs stimulated with MV140/V132 displayed a significant decrease in the mitochondrial membrane potential relative to stimulation with MV140 alone (**Figure 5C**), supporting that the combination of MV140 and V132 induces a metabolic rewiring that enhances glycolysis and lactic fermentation and reduces mitochondrial activity. Supporting these data, MV140/V132-stimulated hmoDCs showed a significantly lower mitochondrial content than hmoDCs stimulated with MV140 alone (**Figure 5D**) as determined by calculating the ratio of mitochondrial DNA (COXII)/nuclear DNA (GAPDH) as described in (45). Next, we studied the potential implication of the transcription factor HIF-1α as a key regulator of glycolysis and other metabolic processes. No changes in the expression of HIF-1α gene were observed after V132 stimulation compared with control cells. However, both MV140- and MV140/V132-activated hmoDCs expressed significantly higher mRNA levels of HIF-1α than excipients- and V132-treated hmoDCs, without significant differences among them (**Figure 5E**). Metabolic reprogramming has been associated with changes in the epigenome *via* histone methylation and acetylation. To assess whether V132, MV140, and MV140/V132 could induce epigenetic reprogramming in hmoDCs, we performed inhibition experiments using the histone methyltransferase inhibitor 5′-methylthioadenosine (MTA) and the histone demethylase inhibitor pargyline. Interestingly, TNF-α, IL-23, and IL-10 production was significantly impaired in the presence of MTA, but preserved in the presence of pargyline after stimulation of hmoDCs with V132, MV140, or MV140/V132 (**Figure 5F**), suggesting that epigenetic modifications are involved in the increased production of the assayed cytokines. An enhanced IL-10 production was observed in the presence of pargyline compared with stimulated cells without the demethylase inhibitor (**Figure 5F**). Cell viability was not significantly affected in any case and only a slight decrease in cell viability was observed for pargyline after V132 or MV140/V132 stimulation (**Supplementary Figure 2**).

### *In Vivo* Sublingual Immunization of BALB/c Mice With MV140/V132 Enhances T Cell Proliferation and Induces Specific Antibodies Against All the Components in the Formulation

**Figure 6:**
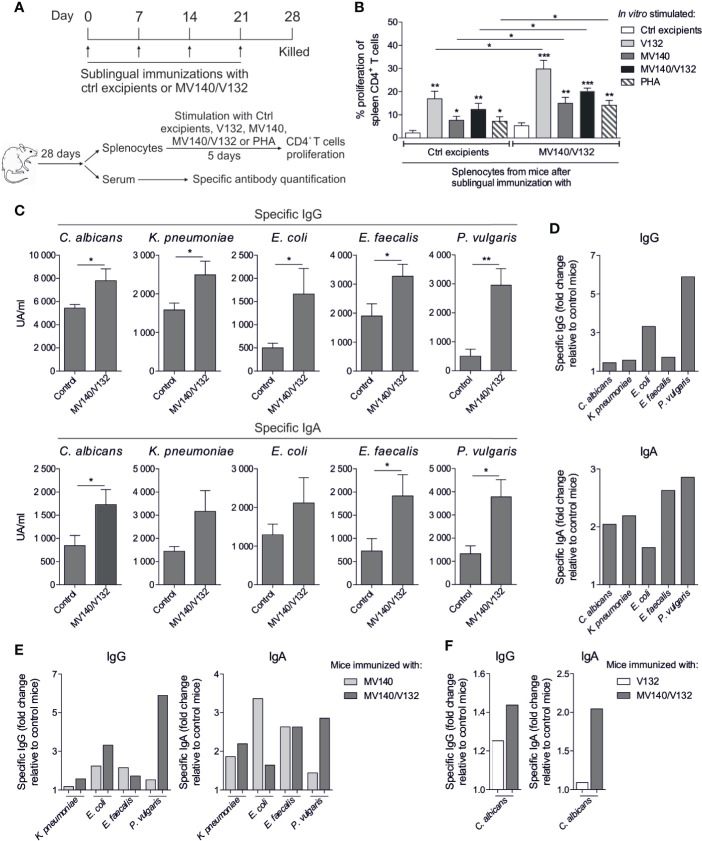
Sublingual immunization of BALB/c mice with MV140/V132 enhances splenic T cell responses and induces specific antibodies against all the components included in the formulation. **(A)** Scheme of the sublingual immunization protocol and analysis of induced systemic responses. **(B)** Proliferation of CFSE-labeled CD4^+^ T cells from splenocytes isolated from mice immunized sublingually with MV140/V132 or control excipients after *in vitro* stimulation with V132, MV140, V140/V132, phytohaemagglutinin (PHA), or control (containing all excipients except the bacteria and yeast). **(C)** Serum IgG and IgA antibodies specific for **(*C*)** *albicans*, *K pneumoniae*, *E coli*, *E faecalis* and *P. vulgaris* from mice immunized with MV140/V132 or with control excipients. **(D)** Fold change of specific IgG and IgA antibodies generated in mice immunized with MV140/V132 relative to control mice. **(E)** Fold change of specific IgG and IgA antibodies for *K pneumoniae*, *E coli*, *E faecalis* and *P. vulgaris* generated in mice immunized with MV140/V132 or MV140 alone relative to control mice. **(F)** Fold change of specific IgG and IgA antibodies for *C albicans* generated in mice immunized with MV140/V132 or V132 alone relative to control mice. Results are mean ± s.e.m. of 7-8 **(B)** and 5-6 **(C–F)** individual mice per condition of one single experiment. Unpaired *t* test, *P < 0.05, **P < 0.01, and ***P < 0.001.

To study the capacity of the different assayed preparations to induce humoral responses *in vivo*, BALB/c mice were sublingually immunized with MV140/V132 or control excipients and the induced T and B cell responses analyzed (**Figure 6A**). Splenic CD4^+^ T cells from mice immunized with MV140/V132 or control excipients showed significantly higher proliferation rates after *in vitro* stimulation with all the assayed stimuli than after *in vitro* stimulation with control excipients (**Figure 6B**). Remarkably, splenic CD4^+^ T cells from mice sublingually immunized with MV140/V132 displayed significantly higher proliferation rates than those from control mice after *in vitro* stimulation with V132, MV140, MV140/V132 or with the non-specific T cell mitogen PHA (**Figure 6B**). Mice immunized with MV140/V132 produced significantly higher levels of specific IgG and IgA against all the components of the vaccine formulation including *C. albicans* (V132) and the individual bacterial components of MV140 (*K. pneumoniae*, *E. coli*, *P. vulgaris* and *E. faecalis*) than control mice (**Figures 6C, D**). As shown in **Figure 6D**, the highest specific IgG responses were against the MV140 gram-negative components *E. coli* and *P. vulgaris* whereas for IgA responses the highest levels were observed for *E. faecalis* and *P. vulgaris*. Interestingly, MV140/V132 immunization induced not only specific IgG1 but also IgG2a against all the components of the vaccine formulation (**Supplementary Figure 3**). As shown in this figure, similar specific IgG1 and IgG2a responses were induced upon MV140/V132 immunization, suggesting also the priming of IFN-γ-producing CD4 T cells driving IgG2a isotype switch. Sublingual immunization with MV140/V132 induced higher IgG responses against *E. coli* and *P. vulgaris* than immunization with MV140 alone whereas only a slight increase for *K. pneumoniae* and a slight decrease for *E. faecalis* were observed (**Figure 6E**). MV140/V132 induced higher IgA responses for *K. pneumoniae* and *P. vulgaris* than immunization with MV140 alone whereas lower IgA levels were detected for *E. coli* without changes for *E. faecalis* (**Figure 6E**). Interestingly, *in vivo* sublingually immunization with MV140/V132 induced higher specific IgG and IgA responses against *C. albicans* than immunization with V132 alone, which was special marked for IgA, thus indicating that the incorporation of MV140 into V132 preparations might well significantly increase the production of mucosal IgA antibodies specific for *C. albicans* compared to V132 alone (**Figure 6F**).

## Discussion

In this study, we demonstrated that the combination of MV140 and V132 at an equal proportion imprints human DCs with the capacity to polarize potent IFN-γ– and IL-17A–producing T cells and FOXP3^+^ regulatory T (Treg) cells. MV140/V132 induces a more potent maturation of DCs and a higher capacity to produce pro-inflammatory cytokines and IL-10 than MV140 or V132 alone. Mechanistically, we showed that MV140/V132 activates MAPK-, NF-κB- and mTOR-mediated signaling pathways in human DCs compared to unstimulated cells, which might well be connected to the induced metabolic and epigenetic reprogramming as key molecular mechanisms driving trained immunity. In particular, MV140/V132-activated hmoDCs enhance glycolytic capacity and reduce mitochondrial activity. MV140/V132 stimulation also leads to histone methylation that enhances the production of TNF-α, IL-23, and IL-10 cytokines by human DCs. *In vivo* sublingual immunization of BALB/c mice with MV140/V132 resulted in enhanced proliferative responses of splenic CD4^+^ T cells not only upon *in vitro* stimulation with the related antigens contained in the vaccine formulation but also upon stimulation with phytohaemagglutinin, a non-specific T cell mitogen, which suggests the induction of trained immunity mechanisms. Remarkably, *in vivo* sublingual immunization with MV140/V132 induces the generation of specific IgG and IgA antibodies against all the components contained in the vaccine formulation. Overall, we report for the first time immunological mechanisms underlying the potential mode of action of a combination of MV140 and V132 as a novel promising trained immunity-based vaccine for GUTIs.

MV140 (Uromune) is a sublingual vaccine for RUTIs prophylaxis that is able to prime human DCs with the capacity to induce Th1-, Th17-, and IL-10–producing T cells (26). Clinical studies have demonstrated that MV140 significantly prevents RUTIs in 80% to 90% of individuals during the year following vaccination (39–43). The reported clinical benefits indicate that sublingual administration of MV140 also reduces the overuse of antibiotics, which not only might reduce the increase in antibiotic-resistances (40), but also avoid microbiota disruption (1, 7, 9–12). Due to the high incidence of RVVCs and the difficulties for their treatment, mainly based on antifungals, new approaches to combat these infections are also fully demanded. Among these alternatives, classical anti-*C. albicans* vaccines based on recombinant proteins or cell wall-derived glycans to induce specific protective antibodies have been developed (46–48). However, up to date no safe and effective antifungal vaccine against RVVCs is still available (25). At this regard, V132 is a mucosal heat-inactivated *C. albicans* vaccine that could represent a suitable strategy to improve treatments for RVVCs. A small pilot study performed by our groups showed that sublingual administration of V132 significantly prevented VVCs recurrence, conferring additional protection to RUTIs when combined at 50% with MV140 in the formulation (unpublished data). Supporting these observations, it was previously reported that intranasal immunization with heat-inactivated *C. albicans* generates protection against *C. albicans* infection in mice and, interestingly, the protective role of this fungal vaccine was higher when mice were immunized with *C. albicans* in combination with *E. coli* toxin as a mucosal adjuvant than the single heat-inactivated fungal administration (49). As RUTIs and RVVCs are related to each other, a polymicrobial formulation combining both MV140 and V132 might well be an alternative approach for the treatment of GUTIs that could improve the efficacy of the vaccination within a single preparation. However, prior to definitive translation into clinical practice further preclinical mechanistic studies are needed. The addition of V132 to MV140 would incorporate *C. albicans* antigens into the vaccine, which could induce antigen-specific responses also against the yeast. In addition, our *in vivo* data indicated that mice immunized with MV140/V132 displayed stronger IgG and IgA responses against *C. albicans* than those immunized with V132 alone, suggesting that MV140 might well enhance the capacity of V132 to induce humoral responses. However, how the combination of V132 and MV140 might regulate mucosal DCs’ function and their capacity to prime adaptive immune responses at the molecular level remained fully elusive.

Our data revealed that MV140/V132 promotes a potent pro-inflammatory cytokine production (TNF-α and IL-23) and higher levels of IL-10 in human DCs compared with individual MV140 or V132 stimulation (26), which are key cytokines also to polarize T cell responses (50, 51). Human DCs activated with MV140/V132 generate potent IFN-γ– and IL-17A–producing T cells that could contribute to eradicate intracellular and extracellular pathogens, respectively (51). MV140/V132 also induces higher frequencies of IL-10–producing T cells and FOXP3^+^ Treg cells than individual MV140 and V132, which are essential to control excessive immune responses, to enhance microbiota protection and tissue repair after long-term antibiotic treatments and play a key role in restoration of healthy immune responses in urogenital infections (50, 52–54). Additionally, *in vivo* sublingual immunization with MV140/V132 induced the generation of significant levels of specific IgG and IgA antibodies against all the components contained in the vaccine formulation, which might well confer protection against infections caused by these specific pathogens (33, 55). It is also noteworthy to highlight that splenic CD4^+^ T cells from mice that were sublingually immunized with MV140/V132 displayed significantly higher proliferation rates than those from control mice not only after *in vitro* stimulation with the vaccine components (either separately or in combination) but also after *in vitro* stimulation with the non-specific T cell mitogen PHA. These data suggest that MV140/V132 immunization might well promote trained immunity mechanisms that enhance both innate and adaptive immune responses against a broad range of pathogens, contained or not in the vaccine formulation, upon new encounters. At this regard, metabolic and epigenetic reprogramming represent well-recognized mechanisms underlying trained immunity in myeloid cells, including DCs (23). The regulation of metabolic rewiring plays an important role in the immunogenic vs tolerogenic properties of DCs (35, 36). For example, the switch to glycolysis and lactic fermentation couple to anabolic metabolism for the biosynthesis needed for cell growth is associated with DCs activation (35, 56). We showed that MV140/V132-activated hmoDCs display enhanced metabolic rate and lactate production while reduced mitochondrial activity, suggesting Warburg effect induction. Supporting this observation MV140/V132 not only induced the rapid activation of immune pathways involved in cytokine and surface marker production such as those mediated by MAPKs or NK-κB but also mTOR-mediated signaling pathways as a central regulator of metabolic reprogramming in hmoDCs (35, 57, 58). mTOR controls numerous cellular processes in DCs to adapt the cellular metabolism to immune functions such as antigen presentation or cytokine production (35, 59). Interestingly, V132 alone and MV140/V132 stimulation induced a higher activation of mTOR signaling pathway than MV140 alone, indicating that V132 significantly contributes to the activation of this key signaling pathway in the induction of innate trained immunity mechanisms. In addition, the activation of the MAPK JNK and p38 are also relevant for the induction of trained immunity in human monocytes (60). Our data show that MV140 is the main driver of p38 and JNK within the MV140/V132 formulation, suggesting also an important contribution of these MV140-induced pathways in the induction of trained immunity. HIF-1α activation is involved in enhanced glycolytic activity and lactate production by the induction of metabolism-related genes (61). MV140 alone and MV140/V132 but not V132 alone enhance HIF-1α mRNA expression levels in hmoDCs. These data suggest that the metabolic reprogramming induced by MV140/V132 might be due to the contribution of both HIF-1α expression, enhanced by bacterial components (MV140) of the preparation (61), and mTOR signaling, induced by *C. albicans* (V132) (31), which together might well drive a more potent glycolytic metabolism. Changes in cellular metabolism could be also accompanied by epigenetic reprogramming, mainly *via* methylation (32, 62). Activation of immune cells induces histone modifications affecting the gene expression patterns (32, 63). The methyltransferase inhibitor MTA but not the histone demethylase inhibitor pargyline abolishes V132-, MV140- and MV140/V132-induced cytokine production in hmoDCs, suggesting that histone methylation is an epigenetic change essential in the mechanism of action of these stimuli (30, 60).

Collectively, MV140/V132-stimulated human DCs activate the Akt/mTOR/HIF-1α axis leading to metabolic and epigenetic reprogramming, which are defining hallmarks of trained immunity. These mechanistic insights might well support the trained immunity effects observed after sublingually immunization of BALB/c mice with MV140/V132 (23, 29–32, 62, 64). In conclusion, the combination of MV140 and V132 in a single vaccine formulation could represent a novel promising TIbV for RUTIs and RVVCs. In the long run and upon proper clinical translations, this study might well pave the way for the development of novel alternative approaches for GUTIs.

## Data Availability Statement

The datasets presented in this article are readily available upon request. Requests to access the datasets should be directed to the corresponding author.

## Ethics Statement

The studies involving human participants were reviewed and approved by the Host Institution UCM. Fully anonymous Buffy Coats from healthy donors were provided by the “Centro de Transfusión de la Comunidad de Madrid”. UCM is perfectly equipped with the adequate biosecurity infrastructure required to carry out the research presented in this proposal. Written informed consent for participation was not required for this study in accordance with the national legislation and the institutional requirements. The animal study was reviewed and approved by Biolab S.L. Animals were maintained in Biolab S.L. under the conditions stabilized by the R.D. 53/2013 and in accordance with the international GLP normative, RD 223/83 and ISO 10993-2:2007. The tests are performed in accordance with official methods, European Pharmacopoeia, USP Pharmacopoeia, OECD Guideline, UNE EN-ISO, and specific methodologies developed for R&D.

## Author Contributions

OP conceived and designed the study. LM-C, CS-O (human experiments), C B-V (technical support for human experiments), and CD-R (mice experiments) performed the experiments. SS-R, JLS, and OP provided the reagents. LM-C, CS-O, CB-V, C.M. D-R, SS-R, JLS, and OP analyzed and discussed the data. OP and L.M-C wrote the paper. All authors contributed to the article and approved the submitted version.

## Funding

This work was supported by grant SAF‐2017‐84978‐R to OP from MINECO, Spain, and by unrestricted grant from Inmunotek under an Art.83 UCM contract to OP. LM-C and CB-V are recipients of FPU predoctoral fellowships from MINECO, Spain. CS-O is recipient of a predoctoral fellowship under an Industrial Doctorate Program (UCM-Inmunotek S.L) sponsored by CAM, Spain.

## Conflict of Interest

OP has received fee for lectures or participation in Advisory Boards from Allergy Therapeutics, Amgen, AstraZeneca, Diater, GSK, Inmunotek SL, Novartis, Sanofi Genzyme, Stallergenes and Regeneron. OP has received research grants from Inmunotek SL and Novartis SL. JS is the founder and CEO of Inmunotek SL.

The remaining authors declare that the research was conducted in the absence of any commercial or financial relationships that could be construed as a potential conflict of interest.

The reviewer SI declared a shared affiliation, with no collaboration, with several of the authors, LC, CO, CV and OP, to the handling editor at the time of review.

## Supplementary Material

Supplementary Figure 1 Production of TGF-β1 and expression of CD86 and HLA-DR in hmoDCs after control excipients, V132, MV140, and MV140/V132 stimulation. **(A)** TGF-β1 production after stimulation of hmoDCs with control excipients, V132, MV140 or MV140/V132 for 18 h quantified in cell-free supernatants by ELISA**. (B)** Flow cytometry representative dot plots for the expression of surface markers HLA-DR and CD86 after stimulation of hmoDCs with control, V132, MV140 and MV140/V132 for 18 h. **(C)** Quantification of the percentage of positive cells and **(D)** Mean Fluorescence Intensity (MFI) values for CD86 after stimulation of hmoDCs. Results are mean ± s.e.m. of 8 **(B, C)** independent experiments. Paired Student *t* test, **P* < 0.05, ***P* < 0.01, and ****P* < 0.001.

Supplementary Figure 2 Percentage of viability for each assayed condition respect to each vehicle control by using trypan blue exclusion. Results are mean ± s.e.m. of 3-4 independent experiments.

Supplementary Figure 3 **(A)** Serum IgG1 and IgG2a antibodies specific for *C. albicans*, *K. pneumoniae*, *E. coli*, *E. faecalis* and *P. vulgaris* from mice immunized with MV140/V132 or with control excipients. **(B)** Fold change of specific IgG1 and IgG2a antibodies generated in mice immunized with MV140/V132 relative to control mice. Results are mean ± s.e.m. of 4-5 individual mice per condition of one independent experiments. Unpaired t test, *P < 0.05.

## Abbreviations

Akt, protein kinase B; APC, allophycocyanin; CLRs, C-type lectin receptors; COXII, cyclooxygenase II; DCs, dendritic cells; FITC, fluorescein isothiocyanate; FTU, Formazan Turbidity Units; GAPDH, glyceraldehyde-3-phosphate dehydrogenase; GUTIs, genitourinary tract infections; HIF-1α, hypoxia inducible factor-1α; hmoDCs, human monocyte-derived DCs; MAPK, mitogen-activated protein kinases; MTA, 5′-deoxy-5′-(methylthio)adenosine; mTOR, mammalian target of rapamycin; NF-κB, nuclear factor-κB; PBMC, peripheral blood mononuclear cells; PBP, polyvalent bacterial preparation; PE, phycoerythrin; PerCP, peridinin-chlorophyll-protein; PHA, phytohaemagglutinin; RUTIs, recurrent urinary tract infections; RVVCs, recurrent vulvovaginal candidiasis; TIbV, trained immunity-based vaccines; TLRs, toll-like receptors; Treg, regulatory T; UTIs, urinary tract infections; VVCs, vulvovaginal candidiasis.
